# Prevalence of chronic pain in children and adolescents with psychiatric conditions

**DOI:** 10.1002/pne2.12100

**Published:** 2023-02-25

**Authors:** Sara Lundqvist, Rajna Knez, Karin Nagy, Salmir Nasic, Nóra Kerekes, Anne‐Katrin Kantzer

**Affiliations:** ^1^ Institute for Neuroscience and Physiology Gothenburg University Gothenburg Sweden; ^2^ Child and Adolescent Psychiatric Clinic Sahlgrenska University Hospital Gothenburg Sweden; ^3^ Department of Paediatrics Skaraborg's Hospital Skövde Sweden; ^4^ FoU Centrum, Skaraborgs Hospital Skövde Sweden; ^5^ Department of Health Sciences University West Trollhättan Sweden; ^6^ Department of Child and Adolescent Psychiatry NU Hospital Group Trollhättan Sweden

## Abstract

The prevalence of pain in children and adolescents with psychiatric conditions is rarely investigated. The aims of the current study were to (a) describe the prevalence of headaches and abdominal pain in children and adolescents with psychiatric conditions, (b) compare the prevalence of pain in children and adolescents with psychiatric conditions with that in the general population, and (c) investigate the associations between pain experience and different types of psychiatric diagnoses. Families with a child aged 6–15 years who had been referred to a child and adolescent psychiatry (CAP) clinic completed the Chronic Pain in Psychiatric Conditions questionnaire. Information about the child/adolescent's psychiatric diagnosis(es) was extracted from the CAP clinic's medical records. The children and adolescents included in the study were divided into diagnostic groups and compared. Their data were also compared with data of control subjects collected during a previous study of the general population. Abdominal pain was more common among girls with a psychiatric diagnosis (85%) than in the matched control population (62%, *p* = 0.031). Children and adolescents with neurodevelopmental diagnoses had a higher prevalence of abdominal pain than children and adolescents with other psychiatric diagnoses. Pain conditions in children and adolescents with a psychiatric diagnosis are common and must be addressed.

## 1 INTRODUCTION

Even though comorbidities are common phenomena in medicine, today's healthcare systems are still fragmented for those with somatic and psychiatric symptoms and diagnoses, including children and adolescents. This fragmentation and the associated lack of a holistic view of child and adolescent care can result in individuals experiencing long‐term and persistent suffering and a greater strain on their families and society.

The mean prevalence of headaches in children has been estimated to be 54.4%–58.4%.^1^, ^2^ In addition, up to 10.5% of all schoolchildren in Europe have frequent abdominal pain.^3^, ^4^ Studies in which children with frequent headaches have been compared to those without headaches have shown that there is a significantly higher prevalence of anxiety and mood disorders in children with frequent headaches.^5^ Depression and anxiety have also been reported as symptoms that frequently occur simultaneously with pain,^5^, ^6^ and clinically significant symptoms of attention‐deficit/hyperactivity disorder (ADHD) and autism spectrum disorder (ASD) have been found to be common in children with chronic debilitating pain.^7^ However, no research has focused on the prevalence of pain in children and adolescents with psychiatric diagnoses in child and adolescent psychiatry (CAP).

Therefore, the current study investigated the prevalence of pain in children and adolescents with psychiatric diagnoses who were in contact with a CAP clinic and compared it to the prevalence of pain in children and adolescents in the general population. The study's hypothesis was that pain is more common in the psychiatric population.

## 2 AIMS

The aims of the current study were to (a) describe the prevalence of headaches and abdominal pain in children and adolescents in contact with a CAP clinic, (b) compare the prevalence of pain in children and adolescents with psychiatric diagnosis(es) with the prevalence in the general population, and (c) investigate the association between pain experience and different types of psychiatric diagnoses.

## 3 METHODS

This was a descriptive exploratory study. From August to November 2018, a questionnaire (the Chronic Pain in Psychiatric Conditions questionnaire, see section 3.2) and an informed consent form for the patient's legal guardians to read and sign were sent to the homes of patients before their first appointment at a CAP outpatient clinic in the region of Västra Götaland. The data collection time frame was limited by the clinic's resources that were used to support the study. The patients and their guardians were asked to complete the questionnaire together during their first clinical appointment. After each patient's appointment, information about their psychiatric diagnosis(es) was collected from their medical records. Data were analyzed using descriptive statistics, and the results related to frequency were compared with those of a previously collected age‐ and sex‐matched general population sample.

### 3.1 Study population

In the current study, questionnaires were distributed to all patients at six different CAP outpatient units in Västra Götaland. Of the 200 distributed questionnaires, 74 were returned (an answer rate of 37%), and 59 (those that were completed in full) were included in the analysis; 15 were excluded because they did not include signed consent from both caregivers. The 59 completed and analyzed questionnaires included responses from 39 boys and 20 girls aged between 6 and 15 years (mean = 12 years). The median age was 11 years for the boys and 13 years for the girls. The questionnaire was completed by the patients and their guardians together.

#### 3.1.1 Semantics

Psychiatric conditions or disorders are defined as “any condition characterized by cognitive and emotional disturbances, abnormal behaviors, impaired functioning, or any combination of these. Such disorders cannot be accounted for solely by environmental circumstances and may involve physiological, genetic, chemical, social, and other factors”.^8^ Neurodevelopmental disorders are included in the classification system used for psychiatric conditions.^8^ In this paper, the term “psychiatric conditions” is used in a general manner to refer to the entire group of different psychiatric and neurodevelopmental conditions.

### 3.2 Psychiatric symptom groups

In Sweden, CAP is a specialized service that only provides care to patients with severe conditions and defined diagnoses. Patients with mild conditions are cared for at general healthcare centers. While the range and combinations of clinical symptoms associated with psychiatric disorders are relatively large, the spectrum of defined psychiatric conditions is relatively small,^8^ and this allowed us to categorize patients into diagnostic groups. We also needed to take into consideration that while participating in the study, the patients might have been undergoing examination and not yet received a Diagnostic and statistical manual of mental health (DSM‐5) diagnosis.^8^

Information about each patient's psychiatric diagnosis was obtained from both the completed questionnaire and the patient's medical record. The patients were then divided into four groups based on general clinical agreement (Table 1). We chose to apply the concept of early symptomatic syndromes eliciting neurodevelopmental clinical examinations (ESSENCE)^9^ to create one group. This concept is being increasingly acknowledged in clinical work globally and includes not only children and adolescents with neurodevelopmental disorders, ADHD and ASD, but also children and adolescents with more general developmental vulnerabilities, such as language delay or behavior problems.^9^ Thus, the ESSENCE Group included patients with ADHD, ASD, and related conditions. The Depression and/or Anxiety Group included patients with depression and/or anxiety disorders. The Eating Disorders Group, including patients with anorexia nervosa, comprised two individuals. The Non‐specified Conditions/Assessment Ongoing Group included patients whose diagnoses were not yet specified and patients under ongoing assessment who may not have received a DSM‐5 diagnosis.

**TABLE 1:** Psychiatric symptom groups.

Number of gender	ESSENCE	Depression and/or Anxiety	Eating disorders	Nonspecified conditions/assessment ongoing	Total
Girls	8	4	1	7	20
Boys	21	6	1	11	39
Total	29	10	2	18	59

Abbreviation: ESSENCE, Early symptomatic syndromes eliciting neurodevelopmental clinical examinations.

It should be noted that the prevalence of diagnoses within this study group is not representative of all CAP patients. Some psychiatric diagnoses were not represented in our sample, and there was a large group with unspecified diagnoses. We chose to focus on comparing the two larger groups with specific diagnoses: the ESSENCE Group and the Depression and/or Anxiety Group. The other two groups (the Eating Disorders and Nonspecified Conditions/Assessment Ongoing Groups) were not included in the comparative analysis of the different groups.

### 3.3 The Chronic Pain in Psychiatric Conditions questionnaire

Due to the lack of a validated instrument containing all the relevant questions, a new questionnaire was created by our research team for the current study. The research team included experienced specialists in CAP and pediatrics and academics with specific knowledge of the population. The questionnaire contains seven questions about headaches and seven questions about abdominal pain. The questions focus on pain intensity, frequency, and severity. One question asks about general health issues, one about medication, one about psychiatric symptoms, and one about the existence of psychiatric diagnoses.

The four selectable responses for the question on pain frequency were as follows: “Rarely/Never,” “Monthly,” “Weekly,” and “Several times a week.” The questionnaire did not capture information on pain duration; therefore, we could not report on “chronic pain” according to the international standard of “At least weekly for at least 3 months.”

Participants used a 10‐point Numeric rating scale (NRS) to describe their pain intensity. Scores between 1 and 3.4 were categorized as mild, between 3.5 and 6.5 as medium, and between 6.6 and 10 as severe pain intensity. To investigate pain severity and its effect on the patient's functioning, we collected information on how the pain affected their presence in school and leisure activities and whether medication was used to treat the pain.

The ROME criteria for functional abdominal pain and the International Classification of Headache Disorders (ICDH) of the International Headache Society (HIS) for headaches were not included in the questionnaire. While professionals are familiar with these criteria, they are difficult for parents to understand and apply. To exclude patients with diagnosed non‐functional pain conditions, an additional question was included on health issues.

### 3.4 Comparison with the general population

To compare the prevalence of pain in our study population with the prevalence of pain in the general population in Sweden, we used the study population from a Swedish study by Brun et al.^10^ as the control group. The control group included 1900 children in grades 3, 6, and 9 from randomly selected schools throughout Sweden. The main aim of Brun et al.'s study was to assess the prevalence of self‐reported pain (i.e., headache, abdominal pain, and musculoskeletal pain) and the perceived health (i.e., problems sleeping and/or often feeling tired, lonely and sad) of the study population. The secondary aim was to examine the co‐occurrence of different pain and health variables. The students (*n* = 1908; grade 3: 255 girls and 305 boys; grade 6: 347 girls and 352 boys; grade 9: 329 girls and 320 boys) retrospectively answered a specially designed questionnaire about their health over the previous 3 months. Half (50%) of the students reported that they had experienced pain, either as headache, abdominal pain or musculoskeletal pain, within the recall period. Gender differences were especially noticeable for headaches, with twice as many girls (17%, *n* = 159) than boys (8%, *n* = 80) reporting that they experienced such pain at least once a week.

### 3.5 Statistics

The questionnaire responses were converted into a database using the Statistical Package for the Social Sciences (SPSS 11.0, version 26). The data were analyzed using both frequencies and cross‐tabulations. The chi‐square test was used to determine the differences in the prevalence of abdominal pain and headaches between the genders, and Fisher's *t*‐test was used to determine the differences between the ESSENCE and Depression and/or Anxiety Groups. The effect size was interpreted using Cramer's *V*.

In the general population comparison, we compared the prevalence of the categories “Often; Every week to always” in Brun et al.^10^ with the categories of “Weekly; Several times a week” in our clinical survey. For all analyses, the level of statistical significance was set at *p* < 0.05. To reduce the risk of a Type I error related to multiple testing, we calculated *p* values adjusted by the Bonferroni correction. The correction was only applied to *p* values that were <0.05 before correction, and the number of performed tests in each table or sub‐analysis was used when the correction was applied.

### 3.6 Ethics

The study was approved by the Regional Ethics Committee at the Medical Faculty, University of Gothenburg, Sweden, Dnr. 313–17. Informed consent was obtained from both parents/responsible guardians of each patient.

## 4 RESULTS

### 4.1 Prevalence and intensity of pain

**FIGURE 1:**
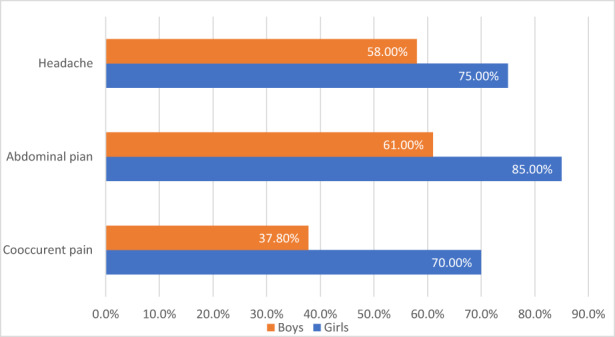
Pain prevalence in the clinical sample.

In the clinical sample, the proportion of girls who reported having headaches (75%) and abdominal pain (85%) at least monthly was higher than the proportion of boys (58% and 61%, *p* = 0.20 and 0.06, Cramer's *V* = 0.169 and 0.251, respectively) (Figure 1). The association between gender and frequency of pain did not reach significance; however, there was a medium to strong association in the case of abdominal pain. Co‐occurring headaches and abdominal pain were reported by 70% of girls and 37.8% of boys (*p* = 0.07; Cramer's *V* = 0.307), showing no significance. However, there was a strong association between combined pain (headache and abdominal pain) and gender.

Most of the patients (51%) specified that their headache intensity was at a medium level. Among the patients with abdominal pain, 55% of the boys specified that the pain intensity was at a medium level and 30% indicated that it was severe. The girls indicated that their pain was more severe: 44% specified that their headache intensity was at a medium level and 66% reported that it was severe. In the patients who had both headaches and abdominal pain, the pain intensity varied greatly.

### 4.2 Prevalence of headaches and abdominal pain in the general population and the clinical sample

**FIGURE 2:**
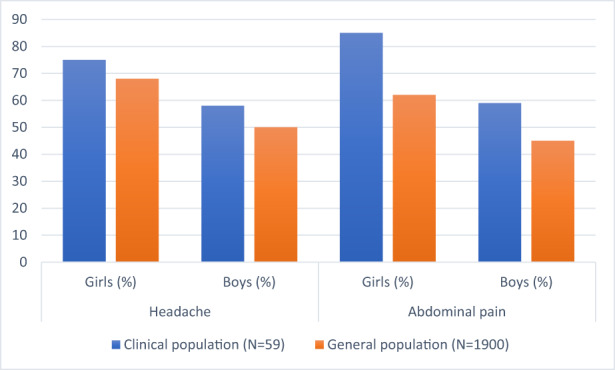
Prevalence of headaches and abdominal pain (at least once a week) in the clinical sample and the general population sample based on Brun et al.^10^

The patients in the clinical sample (both the girls and the boys) were found to have headaches and/or abdominal pain at higher frequencies than children in the general population (based on data from Brun et al).^10^ However, the increased prevalence of pain in children with psychiatric conditions reached statistical significance only in the case of the prevalence of abdominal pain in girls (*p* = 0.032; adjusted *p* = 0.128) (Figure 2; Table 2).

**TABLE 2:** Prevalence of headaches and abdominal pain (at least once a week) in the clinical sample and the general population sample (based on Brun et al.^10^).

	Clinical sample (*N* = 59)	General population (*N* = 1900)	*p*
Headaches			
Girls (%)	75	68	0.54
Boys (%)	58	50	0.33
Abdominal pain			
Girls (%)	85	62	0.032
Boys (%)	59	45	0.06

### 4.3 Prevalence of pain in the psychiatric diagnostic groups

**FIGURE 3:**
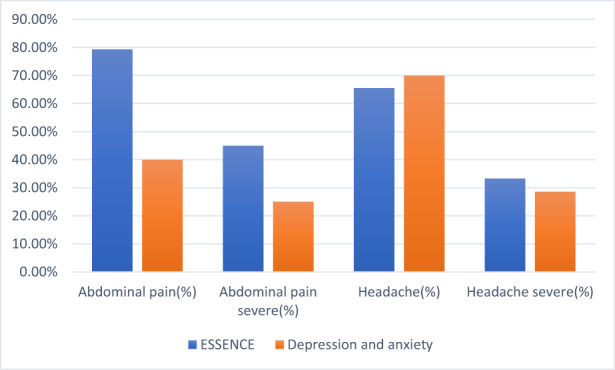
Prevalence of pain in the ESSENCE^9^ and Depression and/or Anxiety Groups.

The prevalence of abdominal pain in the ESSENCE Group was found to be double that of the prevalence in the Depression and/or Anxiety Group (79% and 40%, respectively; *p* = 0.043; adjusted *p* = 0.172). There was no significant difference in the prevalence of headaches detected between the two diagnostic groups (65.5% and 70%, respectively; *p* = 0.28). The prevalence of pain in the two diagnostic groups is summarized in Figure 3.

## 5 DISCUSSION

Studies that have investigated the correlation between pain and the clinical spectrum of psychiatric conditions in children and adolescent populations are rare. This study's findings provide a snapshot of how children and adolescents with psychiatric conditions experience pain and insight into how their pain compares to that of the general population.

The findings show that there is an obvious gender difference: The girls reported having more pain and experiencing the pain as more intense than the boys. There are several discourses about this gender‐based difference. In general, there is a higher prevalence of anxiety and depression among girls, and these conditions have been suggested to be pain predictors and enhancers.^5^, ^6^ Girls enter puberty earlier than boys, so they may experience greater social pressure, which is a risk factor for pain conditions.^11^ Girls also tend to have fewer accidents and less exposure to physical activities (e.g., sports) than boys, which reduces their exposure to acute pain during childhood.^12^ The experience of pain also depends on how people learn to interpret pain; therefore, parents play an important role in teaching young people how to deal with pain,^13^, ^14^ as do coaches, medical staff, and school nurses.^15^

Our results show that abdominal pain was more common than headaches among the boys and the girls in the clinical sample. There is ongoing research on how the brain (the central nervous system) and gut (the enteric nervous system) communicate and depend on each other.^2^ Thus, this type of research may provide new and critical information about the brain–gut relationship that will aid in answering some of the existing questions about the occurrence of abdominal pain in people with psychiatric conditions.

The findings also showed that there was a higher prevalence of abdominal pain in the girls in the CAP population sample than in the girls in the general population sample. This supports the notion that symptoms of anxiety and depression enhance the experience of pain.^5^, ^6^ A possible reason why we did not observe a significant difference in the prevalence of abdominal pain among the boys in the CAP population sample compared to the boys in the general population sample is that the boys in the population were younger than the girls, which could have affected the way they expressed their discomfort and experience of pain.

When we divided the study population into diagnostic groups, we found that patients in the ESSENCE Group (who had mainly neurodevelopmental disorders) experienced more abdominal pain than those in the Depression and/or Anxiety Group. This result indicates that the prevalence of pain is not simply determined by gender and the presence of a psychiatric diagnosis, but that it is also influenced by the type of psychiatric diagnosis. A specific link between pain and neurodevelopmental disorders in children has previously been suggested.^7^ Our results reinforce the need for future studies on pain in children and adolescents with neurodevelopmental disorders.^2^

In this study, we did not investigate other factors that may have been contributing to the pain experienced by the patients, such as social and academic factors. According to our clinical knowledge and previous research,^16^ the stressors of children and adolescents who receive care within CAP are typically related to their social and/or academic environment. Therefore, it is more relevant to discuss such stressors and their relation to pain in individuals who have not developed psychiatric conditions.^4^, ^17^

The findings of this study are important because they provide insight into a clinical population at the time of illness and individuals who are in contact with healthcare services. However, the current study has several limitations. The study population was small, and the response rate was low; therefore, the study did not include a representative sample of the CAP population. This limitation was observed in the prevalence of the different diagnoses in our sample, which were also nonrepresentative in terms of today's clinical picture.

The nonrepresentative gender distribution in our study population further reduces the generalizability of the study. The preschool‐aged patients in CAP are predominantly boys, and the adolescents in CAP are predominantly girls. The actual gender ratio in our CAP clinics is three girls to four boys.^18^ Our sample consisted of twice as many boys with a higher median age than our actual clinical population.

The study sample did, however, contain a reasonable representation of the different diagnoses present in a general outpatient unit. In such units, many children are under ongoing assessment and have not yet received a DSM‐5 diagnosis. The most common diagnoses in a general outpatient unit are ADHD, ASD, depression, and anxiety.^18^ The small number of patients with anorexia nervosa in our study was related to the fact that this condition is usually diagnosed and treated in subspecialty clinics.

Another important limitation that should be taken into account is possible response bias. We could assume that families with children with more pain were more likely to respond because the study would be more relevant for them. This could cause a confounding factor and result in a higher prevalence of pain being reported by the study.

In addition, because of the requirement to inform the study participants with written information and to obtain both legal guardians' consent, we may have missed out on including families who could not read or understand Swedish, as well as children with only one legal guardian or separated parents. In both of these groups, burdensome psychosocial factors are more common, and so too could be the prevalence of functional pain. This limitation would result in the underestimation of pain in the clinical sample.

Regarding the comparison with the general population, a limitation exists because pain was not categorized in exactly the same way in the Chronic Pain in Psychiatric Conditions questionnaire and in Brun et al.'s study.^10^ However, the categorizations were similar. The comparison was further limited by the absence of data on pain intensity in the Brun et al.'s study.

The NRS used in the Chronic Pain in Psychiatric Conditions questionnaire is not validated for children 6 years of age and younger and that is a limitation regarding the pain intensity ratings of the two participants aged 6 years in the population. We included them because of nearby age.

## 6 CONCLUSION

Headaches and abdominal pain without a known underlying cause—experienced separately or together—are common symptoms in children and adolescents with psychiatric diagnoses. The current study has shown that abdominal pain may be more common among girls with psychiatric diagnoses than it is in children and adolescents in the general population. Among the participants with psychiatric diagnoses, those in the ESSENCE Group were found to have the highest prevalence of abdominal pain. Our findings reinforce the importance of addressing pain in psychiatric care and psychiatric symptoms in somatic care.

## Data Availability

All available data are analysed and published in this article.

## References

[1] Abu‐Arafeh I , Razak S , Sivaraman B , Graham C . Prevalence of headache and migraine in children and adolescents: a systematic review of population‐based studies. Dev Med Child Neurol. 2010;52(12):1088‐1097. 20875042 10.1111/j.1469-8749.2010.03793.x

[2] Kerekes N , Lundqvist S , Hjalmarson ES , Torinsson Naluai Å , Kantzer AK , Knez R . The associations between ADHD, pain, inflammation, and quality of life in children and adolescents‐a clinical study protocol. PLoS One. 2022;17(9):e0273653. 36083951 10.1371/journal.pone.0273653 PMC9462574

[3] Korterink JJ , Diederen K , Benninga MA , Tabbers MM . Epidemiology of pediatric functional abdominal pain disorders: a meta‐analysis. PLoS One. 2015;10(5):e0126982. 25992621 10.1371/journal.pone.0126982 PMC4439136

[4] Luntamo T , Sourander A , Rihko M , et al. Psychosocial determinants of headache, abdominal pain, and sleep problems in a community sample of Finnish adolescents. Eur Child Adolesc Psychiatry. 2012;21:s301‐s313. 10.1007/s00787-012-0261-1 22350133

[5] Pavone P , Rizzo R , Conti I , et al. Primary headaches in children: clinical findings on the association with other conditions. Int J Immunopathol Pharmacol. 2012;25(4):1083‐1091. 23298498 10.1177/039463201202500425

[6] Piwowarczyk P , Kaczmarska A , Kutnik P , et al. Association of gender, painkiller use, and experienced pain with pain‐related fear and anxiety among university students according to the fear of pain questionnaire. Int J Environ Res Public Health. 2021;18(8):4098. doi:10.3390/ijerph18084098 33924523 PMC8068817

[7] Lipsker CW , Bölte S , Hirvikoski T , Lekander M , Holmström L , Wicksell RK . Prevalence of autism traits and attention‐deficit hyperactivity disorder symptoms in a clinical sample of children and adolescents with chronic pain. J Pain Res. 2018;11:2827‐2836. doi:10.2147/JPR.S177534 30519085 PMC6235327

[8] American Psychiatric Association . Diagnostic and Statistical Manual of Mental Disorders. 5th ed. American psychiatric association; 2013.

[9] Gillberg C . The ESSENCE in child psychiatry: early symptomatic syndromes eliciting neurodevelopmental clinical examinations. Res Dev Disabil. 2010;31(6):1543‐1551. doi:10.1016/j.ridd.2010.06.002 20634041

[10] Brun GM , Sarrtok T , Engström LM . Prevalence and co‐occurrence of self‐rated pain and perceived health in school‐children: age and gender differences. Eur J Pain. 2007;11(2):171‐180. 16542860 10.1016/j.ejpain.2006.02.006

[11] Picavet HSJ , Gehring U , Van Haselen A , et al. A widening gap between boys and girls in musculoskeletal complaints, while growing up from age 11 to age 20 ‐ the PIAMA birth cohort study. Eur J Pain. 2021;25(4):902‐912. doi:10.1002/ejp.1719 33405263 PMC8048429

[12] Räisänen A , Kokko S , Pasanen K , et al. Prevalence of adolescent physical activity‐related injuries in sports, leisure time, and school: the National Physical Activity Behaviour Study for children and adolescents. BMC Musculoskelet Disord. 2018;19(1):58. doi:10.1186/s12891-018-1969-y 29448928 PMC5815200

[13] Miller MM , Meints SM , Hirsh AT . Catastrophizing, pain, and functional outcomes for children with chronic pain: a meta‐analytic review. Pain. 2018;159(12):2442‐2460. 30015710 10.1097/j.pain.0000000000001342 PMC6237640

[14] Noel M , Alberts N , Langer SL , Levy RL , Walker LS , Palermo TM . The sensitivity to change and responsiveness of the adult responses to children's symptoms in children and adolescents with chronic pain. J Pediatr Psychol. 2016;41(3):350‐362. doi:10.1093/jpepsy/jsv095 26493601 PMC5896805

[15] Goubert L , Vlaeyen JWS , Crombez G , Kenneth DC . Learning about pain from others: an observational learning account. J Pain. 2011;12(2):167‐174. doi:10.1016/j.jpain.2010.10.001 21111682

[16] Hultman O , Broberg A . Family violence and other potentially traumatic interpersonal events among 9‐ to 17‐year‐old children attending an outpatient psychiatric clinic. J Interpers Violence. 2016;31(18):2958‐2986. doi:10.1177/0886260515584335 25917005

[17] Roth‐Isigkeit A , Thyen U , Stöven H , Schwarzenberger J , Schmucker P . Pain among children and adolescents: restrictions in daily living and triggering factors. Pediatrics. 2005;115(2):e152‐e162. doi:10.1542/peds.2004-0682 15687423

[18] Sveriges kommuner och landsting, avdelningen för vård och omsorg . Uppdrag psykisk hälsa. Kartläggningen psykiatri i siffror.https://www.uppdragpsykiskhalsa.se/psykiatrin‐i‐siffror/ 2022‐05‐22.

